# In Vitro Bioaccessibility and Antioxidant Activity of Phenolic Compounds in Coffee-Fortified Yogurt

**DOI:** 10.3390/molecules27206843

**Published:** 2022-10-12

**Authors:** Ahmed Helal, Alice Cattivelli, Angela Conte, Davide Tagliazucchi

**Affiliations:** 1 Department of Food and Dairy Sciences and Technology, Damanhour University, Damanhour 22516, Egypt; 2 Department of Life Sciences, University of Modena and Reggio Emilia, Via Amendola 2-Pad. Besta, 42100 Reggio Emilia, Italy

**Keywords:** functional food, hydroxycinnamic acids, polyphenols, radical scavenging activity, bioaccessibility

## Abstract

Yogurt is considered one of the most popular and healthy dairy products, and has been exploited as a delivery matrix for phenolic compounds. In this study, coffee powder was added to yogurt as a functional ingredient to produce coffee-fortified yogurt. Total phenolic compounds, antioxidant activity and individual hydroxycinnamic acids have been identified and quantified through mass spectrometry. The results from coffee-fortified yogurt were compared with fermented coffee and plain yogurt. Coffee-fortified yogurt had higher total phenolic content and antioxidant activity compared to plain yogurt. However, the total phenolic compounds found in coffee-fortified yogurt represented only 38.9% of the original content in coffee. Caffeoylquinic acids were the most abundant phenolic compounds in coffee. Fermented coffee and coffee-fortified yogurt displayed lower amounts of individual phenolic compounds with respect to coffee (69.8% and 52.4% of recovery, respectively). A protective effect of the yogurt matrix on total and individual coffee phenolic compounds has been observed after in vitro digestion, resulting in a higher bioaccessibility in comparison with digested fermented coffee. Moreover, coffee-fortified yogurt showed the highest antioxidant values after digestion. These findings clearly demonstrate that coffee-fortified yogurt can be considered a significant source of bioaccessible hydroxycinnamic acids, besides its health benefits as a fermented dairy product.

## 1. Introduction

The term “functional food” is used to describe foods that, in addition to their basic nutritional role, may have beneficial effects on human health by improving physiological responses and/or lowering the risk of chronic diseases [1]. In the last few years, the search for a healthier and more sustainable diet has become increasingly popular throughout the world. The demand for healthy products continues to increase, in line with the number of people who are concerned about their health and well-being.

Yogurt, the most popular fermented dairy product, is a good example of a functional food. It is manufactured from milk fermented by the symbiotic action of *Streptococcus thermophilus* and *Lactobacillus bulgaricus,* and is appreciated for its nutritional attributes related to high protein content, and the presence of some vitamins (such as folic acid, vitamin A and some vitamins from the B- group) and minerals [2,3,4]. Several health benefits have been related to yogurt consumption, including strengthening the immune system, improvement of the digestive function, and protection against colon cancer and *Helicobacter pylori* infection [5]. Moreover, diverse in vivo studies have suggested that yogurt consumption may have some protective effects against the onset of chronic diseases, especially cardiovascular diseases [6,7].

Frequently, to improve the aroma, taste, and consistency of the yogurt, fruits, sweeteners, and flavors are added. The presence, the type and the healthy properties of these flavors are carefully considered by consumers in their choices [8,9]. Therefore, the number of dairy products in the market has increased in the past decade, with a wide variety of products relative to flavored yogurt. Furthermore, the addition of traditional herbs and/or vegetable foods or extracts allows the preparation of yogurt with enhanced healthy properties thanks to the presence of phenolic compounds [3,8,10,11,12,13,14,15,16,17,18,19].

Coffee is one of the most popular beverages in the world because of its widely appreciated flavor and aroma, as well as for its stimulant effects and the proven health benefits [20,21,22]. Epidemiological studies have correlated regular coffee drinking with a reduced risk of several chronic diseases such as heart diseases, Parkinson’s disease, diabetes, and several typologies of cancer [23,24,25,26]. As reported by several investigations, chlorogenic acids, which represent the major coffee phenolic compounds, have been proposed as the primary factor responsible for the health effects of coffee, especially in the prevention of cardiovascular disease and diabetes [27,28]. Due to such positive health effects, coffee has the ability to impart specific functional qualities to yogurt as a natural food additive.

Tagliazucchi et al. [29] reported that bioaccessibility is one of the most important factors influencing the positive activity of phenolic compounds in terms of health benefits. Bioaccessibility is described as the amount of a compound released from the food matrix that persists under stomach and intestinal conditions and can be deemed available for absorption in the gastro-intestinal tract [30]. This last point is critical because only those compounds that are released from the food matrix and remain stable in the gastro-intestinal environment have the potential to be accessible and exert their effects on the digestive system, or systematically.

The yogurt matrix appears to be an effective vehicle for the delivery of phenolic compounds present in vegetable foods. According to Chouchouli et al. [31], low pH increases the stability of phenolic compounds when they are stored, whereas the presence of proteins or large peptides and fat should help to maintain the integrity of phenolic compounds during digestion, thereby increasing their bioaccessibility [8,32,33].

Therefore, the goal of this research was to incorporate coffee into proteinaceous foods such as yogurt in order to boost their phenolic component content and to produce a more nutritious food product. Furthermore, we sought to determine the bioaccessibility of phenolic compounds and antioxidant activity during simulated gastro-intestinal digestion.

## 2. Results and Discussion

### 2.1. Total Phenolic Content, Individual Phenolic Compounds and Antioxidant Properties of Coffee, Fermented Coffee, Plain Yogurt and Coffee-Fortified Yogurt

To investigate the effects of milk and the fermentation process on coffee phenolic compounds, a coffee decoction was prepared in the same way as coffee-fortified yogurt, but with water instead of milk. Then, the coffee sample was subjected to the same fermentation process as for yogurt samples, obtaining a fermented coffee. Both coffee and fermented coffee as well as plain yogurt and coffee-fortified yogurt were tested for the total phenolic content, the individual phenolic profile, and the antioxidant properties. As reported in the Materials and Methods section, the analysis was carried out on the supernatant of the samples obtained after centrifugation.

**Figure 1:**
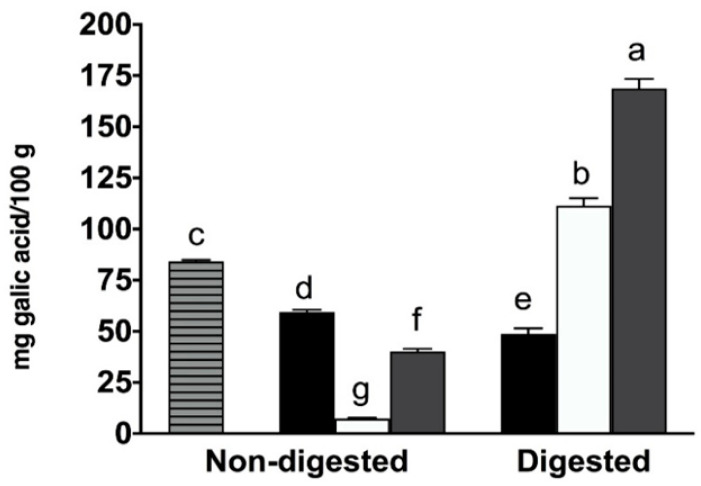
Total phenolic content measured in the supernatants after centrifugation and expressed as mg of gallic acid equivalent/100 g of sample of coffee (striped) as well as before and after in vitro digestion of fermented coffee (black), plain yogurt (white) and coffee-fortified yogurt (grey). Values are means of three independent replicates ± standard deviation (SD). Different letters indicate significantly different values (*p* < 0.05).

As shown in Figure 1, the total amount of phenolic compounds extracted in the coffee sample was 84.15 ± 0.79 mg of gallic acid/100 g of coffee. Moreover, there was a significant decrease (*p* < 0.05) in total phenolic content after the fermentation of the coffee, which resulted in a 29.4% reduction, plausibly due to the presence of starter lactic acid bacteria. Recently, Makarewicz et al. [34] highlighted the mechanism of interaction between phenolic compounds and bacteria. They demonstrated that phenolic compounds may bind to a variety of proteins from bacteria, such as adhesins, cell envelope enzymes and cell envelope transport proteins, and these formed complexes may negatively affect phenolic compounds’ recovery and bioactivity.

As reported in Figure 1, plain yogurt displayed a low reactivity with the Folin–Ciocalteau reagent (7.33 ± 0.40 mg of gallic acid/100 g of yogurt), mainly due to amino acids (such as tyrosine), peptides and milk proteins [8,35,36]. Compared to plain yogurt, the addition of coffee resulted in a significant increase (*p* < 0.05) in total phenolic compounds in the coffee-fortified yogurt sample (Figure 1). This agrees with previous studies on yogurt fortified with different phenolic compound sources, such as hazelnut, grape, coffee, tea, tomato pomace and cinnamon [8,13,17,37,38,39]. However, the amount of total phenolic compounds in coffee-fortified yogurt (40.10 ± 1.34 mg of gallic acid/100 g of coffee-fortified yogurt) was significantly lower (*p* < 0.05) than in coffee (84.15 ± 0.79 mg of gallic acid/100 g of coffee) and fermented coffee (59.43 ± 1.08 mg of gallic acid/100 g of fermented coffee), with a calculated recovery of 43.8% and 67.5%, respectively. The decrease in total phenolic compounds in coffee-fortified yogurt was more evident when the contribution of plain yogurt was considered, resulting in a value of 32.77 ± 1.28 mg of gallic acid/100 g of coffee-fortified yogurt, and a recovery of 38.9% with respect to coffee.

The presence of milk proteins, which can bind and precipitate coffee phenolic compounds, may be responsible for this decrease in total phenolic compounds in coffee-fortified yogurt. Due to the presence of large hydrophobic regions, milk proteins can bind a wide variety of small ligands, albeit with different degrees of affinity. It has been shown that various interactions, such as hydrogen bonds as well as ionic and hydrophobic interactions, play a significant role in protein–phenolic interactions [40]. Moreover, β-casein, α-caseins, κ-casein, and whey proteins have previously been found to be able to interact with coffee phenolic compounds with a different affinity depending on ionic strength, pH, temperature, and other environmental and food processing factors [41]. Furthermore, several studies have shown that the interactions between caseins and coffee hydroxycinnamic acids were the strongest at acidic pH levels, such as those found in yogurt during fermentation [42,43]. Similarly, previous works found a decrease in total phenolic content in yogurts with added strawberry, tomato pomace, cinnamon, or pomegranate juice compared to the control samples without milk addition [8,17,18,44].

Individual phenolic compounds in coffee, fermented coffee and coffee-fortified yogurt samples were identified and quantified using LC-ESI-IT-MS/MS analysis, and the results are displayed in Table 1.

Three caffeoylquinic acids (3-*O*-caffeoylquinic acid, 4-*O*-caffeoylquinic acid and 5-*O*-caffeoylquinic acid), four caffeoylquinic acid-glycoside isomers, three di-caffeoylquinic acids (3,4-*O*-di-caffeoylquinic acid, 3,5-*O*-di-caffeoylquinic acid and 4,5-*O*-di-caffeoylquinic acid) and three feruloylquinic acids (3-*O*-feruloylquinic acid, 4-*O*-feruloylquinic acid and 5-*O*-feruloylquinic acid) have been identified and quantified.

**Figure 2:**
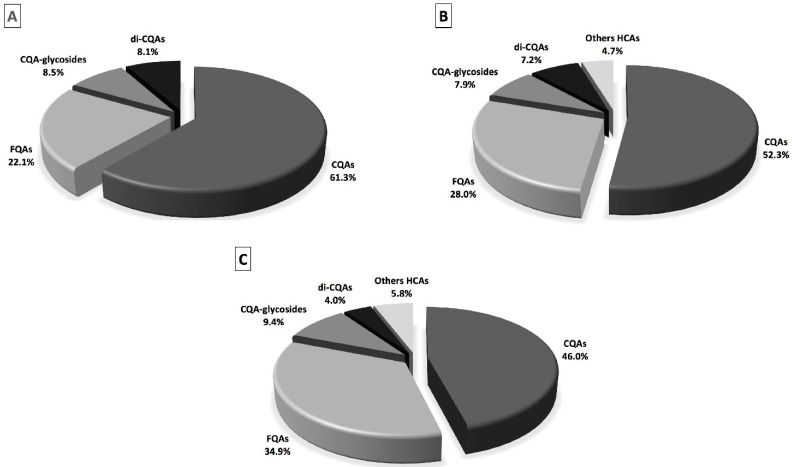
Percentages of individual hydroxycinnamic acids in the different treatments. The percentage values refer to the percentages of each hydroxycinnamic acid with respect to the total hydroxycinnamic acids identified in the sample. Coffee (**A**), fermented coffee (**B**) and coffee-fortified yogurt (**C**). CQAs: caffeoylquinic acids; FQAs: feruloylquinic acids; CQAs-glycosides: caffeoylquinic acid-glycoside; di-CQAs: di-caffeoylquinic acids; other HCAs: other hydroxycinnamic acids.

In coffee, caffeoylquinic acids were the most representative identified phenolic compounds (61.3% of total phenolic compounds), followed by feruloylquinic acids, caffeoylquinic acids-glycosides and di-caffeoylquinic acids, which represented 22.1%, 8.5% and 8.1% of the total phenolic compounds, respectively (Figure 2A). Among the individual phenolic compounds, 3-*O*-caffeoylquinic and 5-*O*-caffeoylquinic acids were the most prevalent hydroxycinnamic acids detected, accounting for 28.7% and 27.5% of the total phenolic compounds, respectively. The reported data agree with previous studies suggesting that 3-*O*-caffeoylquinic and 5-*O*-caffeoylquinic acids accounted for the highest proportion of total phenolic compounds observed in various coffees [33,45,46,47]. On the contrary, di-caffeoylquinic acids are less soluble than caffeoylquinic acids; hence, they are released more slowly during water extraction [48].

The fermentation of coffee resulted in a significant decrease in all the classes of phenolic compounds (Table 1). Feruloylquinic acids was the class with the highest recovery (88.4%), followed by caffeoylquinic acid-glycosides (65.2% of recovery) and di-caffeoylquinic acids (61.5% of recovery), whereas caffeoylquinic acids showed the lowest recovery in fermented coffee (59.6%). Consequently, the percentage incidence of caffeoylquinic acids decreased with respect to coffee, whereas that of feruloylquinic acids increased (Figure 2A,B). Considering all the phenolic compounds identified by mass spectrometry, the decrease in the fermented coffee sample with respect to coffee was 30.2%. Regarding the individual phenolic compounds belonging to the most representative classes (caffeoylquinic and feruloylquinic acids), it can be observed that the amounts of 3-*O*- and 5-*O*-caffeoylquinic acids, as well as of 3-*O*- and 5-*O*-feruloyquinic acids, significantly decreased in fermented coffee with respect to coffee, whereas the concentration of 4-*O*-caffeoylquinic acid as well as of 4-*O*-feruloyquinic acid increased. These results indicate that during coffee fermentation, trans-esterification reactions took place. Previously, Deshpande et al. [49] ascertained acyl migration under acidic conditions from 3-*O*-caffeoylquinic acid to 4-*O*-caffeoylquinic acid, whereas the 5-*O*-acyl isomer was found to be stable at low pH values and did not show any acyl migration product. Therefore, the observed decrease in 3-*O*- and 5-*O*-caffeoylquinic acids’ concentrations after coffee fermentation may be a consequence of isomerization reactions, degradation to some non-identified compounds, as well as binding to bacterial proteins. The same conclusions may be drawn in the case of feruloylquinic acids.

In addition, some new compounds, e.g., two isomers of caffeoylshikimic acid, were detected after coffee fermentation (Table 1). The release of phenolic compounds from coffee after fermentation may be due to the hydrolysis catalyzed by bacterial enzymes of coffee macromolecules such as melanoidins [50]. It is well known that coffee melanoidins contain in their structure different phenolic acids, which may be released by the enzymatic action of bacteria during fermentation [51].

The decrease in coffee hydroxycinnamic acids was more evident in the coffee-fortified yogurt sample, especially for caffeoylquinic acids (Table 1). For this class of phenolic compounds, the recovery values in coffee-fortified yogurt were less than 40%. The greater decrease in the amounts of caffeoylquinic acids in the coffee-fortified yogurt sample may be due to the binding between these hydroxycinnamic acids and milk caseins during yogurt preparation, since these compounds showed high affinity versus milk caseins [33]. As shown in Figure 2C, in coffee-fortified yogurt, there was an increase in the percentage incidence of feruloylquinic acids, suggesting that these phenolic compounds may have less affinity than milk proteins, as previously suggested [33]. Therefore, the different binding affinities between phenolic compounds and milk proteins may explain the differences in the recovery among coffee hydroxycinnamic acids [52]. As observed in fermented coffee, in the coffee-fortified yogurt sample, some new compounds also appeared after fermentation (caffeoylshikimic acid isomer 2 and caffeoyl hexose), whereas others increased in concentration (such as 4-*O*-feruloylquinic acid).

**Figure 3:**
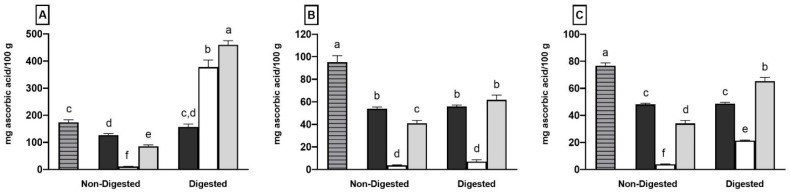
Antioxidant activity of coffee (striped), fermented coffee (black), plain yogurt (white) and coffee-fortified yogurt (grey) before and after in vitro digestion determined by ABTS (**A**), DPPH (**B**) and FRAP (**C**) assays. Values are means of three independent replicates ± standard deviation (SD). Different letters indicate significantly different values (*p* < 0.05).

Figure 3 displays the data of the antioxidant properties of the different samples determined by three different assays.

Coffee fermentation determined a decrease in the antioxidant activity in all the assays with respect to the coffee sample, according to the reduction in total phenolic compounds, as shown in Figure 1. This last sample exhibited the highest antioxidant activity values out of all the assays (Figure 3), whereas the lowest values were found in the plain yogurt sample, whose antioxidant activity was mostly due to the proteolytic activity of the starter lactobacilli during yogurt production leading to the formation of peptides and/or amino acids with antioxidant activity [4,53]. As illustrated in Figure 3A–C, the coffee-fortified yogurt sample had a higher antioxidant activity in all three assays than plain yogurt samples (*p* < 0.05). This increase in the fortified yogurt compared to the plain one is a result of the presence of coffee phenolic compounds. Previous studies also found that yogurt fortified with phenolic compounds has increased antioxidant activity [13,37,54]. However, the recorded values of scavenging activity in the supernatant of the coffee-fortified yogurt sample were significantly lower (*p* < 0.05) with respect to coffee and fermented coffee in all the assays, according to the Folin–Ciocalteau data. This decrease was primarily due to the binding of coffee phenolic compounds with milk proteins during yogurt preparation. Furthermore, previous studies have also found that milk proteins had a negative effect on the antioxidant activity of phenolic compounds when the expected value was compared to the observed one [55,56].

### 2.2. Effect of In Vitro Digestion on Bioaccessibility of Total and Individual Phenolic Compounds and Antioxidant Activity

To exert their activity at a systemic level, phenolic compounds have to be bioavailable, i.e., they have to be released from the food matrices, absorbed at the intestinal level and reach the target organs [57]. The bioavailability of hydroxycinnamic acids is generally considered low, since they are only slightly absorbed at the intestinal level and most of them reach the colon, where they are metabolized by the gut microbiota [58,59]. Nevertheless, hydroxycinnamic acids may exert their biological activity in the gastro-intestinal tract before absorption, where they may act as anti-inflammatory and anti-proliferative compounds, protecting, for example, against inflammatory bowel disease and colon cancer [60,61]. In this last case, hydroxycinnamic acids have to be bioaccessible (rather than bioavailable), which means that they need to be released from the food matrices and stable during gastro-intestinal digestion. Therefore, to figure out the bioaccessibility of coffee hydroxycinnamic acids and the effect of the food matrix, the different preparations were submitted to an in vitro gastro-intestinal digestion protocol.

Figure 1 shows the changes in total phenolic content in the differently prepared samples before and after in vitro digestion. At the end of the digestion, the total phenolic compounds in fermented coffee slightly but significantly decreased (*p* < 0.05) from 59.43 ± 1.08 to 48.80 ± 2.67 mg of gallic acid/100 g of fermented coffee. However, the bioaccessibility index calculated based on the initial amount of total phenolic compounds (coffee sample) was 58.0% (Figure 1). At the end of the digestion process, the coffee-fortified yogurt sample showed a total phenolic compound content of 168.80 ± 4.64 mg of gallic acid/100 g of the coffee-fortified yogurt, which resulted in a value of 57.44 mg/100 g when corrected for the contribution of plain yogurt (111.36 ± 3.71 mg of gallic acid/100 g of yogurt). The corrected value of coffee-fortified yogurt after digestion was compared with the initial total phenolic content in coffee, and this ratio was used to determine the total phenolic bioaccessibility index. The coffee-fortified yogurt sample had a bioaccessibility index of 68.3%, which is significantly higher than that observed in the digested fermented coffee.

As reported in Table 1, the majority of the individual hydroxycinnamic acids decreased after the in vitro digestion of fermented coffee, resulting in a bioaccessibility index of 25.2% when calculated considering the initial amounts of total hydroxycinnamic acids in unfermented coffee. Regarding the caffeoylquinic acids, there was a significant decrease in their amount after the in vitro digestion of fermented coffee, with a calculated bioaccessibility index of 14.5%. This decrease was noted among all the individual caffeoylquinic acids. On the other hand, feruloylquinic acids showed the highest recorded bioaccessibility index of 74.0% after the in vitro digestion of fermented coffee. Tagliazucchi et al. [33] observed a greater stability of feruloylquinic acids during gastro-intestinal digestion in instant coffee, finding higher bioaccessibility for 4-*O*- and 5-*O*-feruloylquinic acids with respect to 3-*O*-feruloylquinic acid. Caffeoylquinic acid-glycosides and di-caffeoylquinic acids were not detected after the in vitro digestion of fermented coffee. Previous studies showed that di-caffeoylquinic acids were more affected by gastro-intestinal digestion, since the bioaccessibility index was low or even undetectable [33,47].

The influence of the interaction between phenolic compounds and digestive enzymes, and the influence of pH on the decrease in hydroxycinnamic acids observed during in vitro digestion, has been previously reported, and may partially explain the observed loss of these compounds after fermented coffee digestion [33,62,63,64,65]. Moreover, hydroxycinnamic acids can be easily oxidized to the corresponding quinone at pH levels above 7, which in turn can react with proteins or may undergo polymerization [66]. The oxidation and polymerization of hydroxycinnamic acids could be potential pathways of loss of these compounds during the in vitro digestion of fermented coffee.

As reported in Table 1, coffee fermentation in the presence of milk as in the coffee-fortified yogurt sample resulted in a protective effect on hydroxycinnamic acids’ stability and release after in vitro digestion. The total amount of hydroxycinnamic acids in the coffee-fortified yogurt sample was significantly higher than that of fermented coffee after in vitro digestion (13.98 ± 0.16 and 9.46 ± 0.09 μmol/100 g, respectively), resulting in a higher bioaccessibility index (37.3% and 25.2%, respectively). Similarly to the fermented coffee sample, caffeoylquinic acid-glycosides and di-caffeoylquinic acids were not detected in the coffee-fortified yogurt sample submitted to in vitro digestion. Once again, feruloylquinic acids showed the highest bioaccessibility index between the different hydroxycinnamic acids (127.1%), whereas the bioaccessibility index of caffeoylquinic acids was lower (15.0%) and similar to that observed after fermented coffee digestion. Therefore, the described results highlight that the food matrix (i.e., milk and yogurt) and its components may improve total phenolic and hydroxycinnamic acids’ stability and bioaccessibility after in vitro digestion.

The protective effect of the yogurt matrix on coffee phenolic compounds during in vitro digestion may be a consequence of the observed binding between coffee phenolic compounds and milk proteins during yogurt preparation. The binding with caseins may prevent the interaction of coffee phenolic compounds with digestive enzymes, and/or may protect coffee phenolic compounds from oxidative reactions. After that, the hydrolysis of the milk protein during digestion may favor the release of bound coffee phenolic compounds, resulting in an improved bioaccessibility index.

Previous studies found that the inclusion of dairy matrices greatly increased phenolic compounds’ stability and bioaccessibility during digestion, because of the interaction between milk proteins and phenolic compounds [8,56,67,68]. Therefore, milk proteins can be considered a carrier for the delivery of phenolic compounds through the gastro-intestinal tract, preventing their binding with digestive enzymes and protecting them from oxidation processes. Afterwards, phenolic compounds may be slowly and continuously released from milk proteins as hydrolysis proceeds, resulting in an increased stability and bioaccessibility.

The effect of in vitro digestion on the antioxidant activity of the different preparations is shown in Figure 3. No significant differences were found for the fermented coffee in terms of the antioxidant activity values determined before and after in vitro digestion in all three assays.

On the contrary, the antioxidant activity of the plain yogurt sample increased after in vitro digestion, reaching values of 377.72 ± 26.44, 7.07 ± 1.58 and 21.40 ± 0.41 mg ascorbic acid/100 g of yogurt for the ABTS, DPPH and FRAP assays, respectively.

The release of antioxidant peptides and amino acids, encrypted in the milk protein sequences, during in vitro digestion, may explain the observed increase in the antioxidant activity of plain yogurt. Furthermore, it has been previously shown that the peptides from caseins with high levels of histidine, tryptophan, methionine, and tyrosine are released during the in vitro digestion of milk or dairy products, showing excellent antioxidant abilities [10,69,70].

The highest increase in antioxidant activity after plain yogurt digestion was found in the ABTS assay (about 34.5 times), followed by the FRAP assay (about 5.4 times) and DPPH assay (about 1.9 times). These results suggest that antioxidant peptides and/or amino acids were more reactive in the ABTS assay with respect to the other two antioxidant activity assays.

The increase in the antioxidant activity after the in vitro digestion of coffee-fortified yogurt was more pronounced than that observed for plain yogurt in all the different assays, resulting in values of 460.04 ± 15.27, 61.87 ± 4.19 and 65.39 ± 2.68 mg ascorbic acid/100 g of coffee-fortified yogurt for the ABTS, DPPH and FRAP assays, respectively. Similar results have been observed by Bertolino et al. [13] and Helal and Tagliazucchi [8], as they found an increase in the antioxidant activity of coffee silverskin-fortified yogurt and cinnamon-fortified yogurt after in vitro digestion, with respect to the corresponding yogurt sample.

The antioxidant activity of the coffee-fortified yogurt sample should be the result of the contribution of the coffee phenolic compounds and milk components released during in vitro digestion. However, the antioxidant activity of the in vitro digested coffee-fortified yogurt sample was significantly lower than the sum of the antioxidant activity of digested plain yogurt and fermented coffee, especially in the ABTS and FRAP assays, despite the higher amounts of phenolic compounds released from the coffee-fortified yogurt during digestion with respect to fermented coffee. To gain more information about this unexpected effect, the hydrolysis degree of milk proteins during the digestion of plain yogurt and coffee-fortified yogurt samples was determined. A significant decrease (*p* < 0.05) in milk proteins hydrolysis in the coffee-fortified yogurt sample was noticed compared to plain yogurt (12.32 ± 0.33 and 13.76 ± 0.70 mmol of leucine/100 g of sample in coffee-fortified yogurt and plain yogurt, respectively), which may result in a lower amount of antioxidant peptides and/or amino acids being released in coffee-fortified yogurt with respect to plain yogurt.

The decrease in milk proteins hydrolysis observed in the coffee-fortified yogurt sample may be due to the inhibitory effect of coffee hydroxycinnamic acids on intestinal proteases, such as trypsin, or to their binding to milk proteins that sterically hamper the access of proteases to the peptidic bonds, resulting in an improved protein stability during in vitro digestion [71].

## 3. Materials and Methods

### 3.1. Materials

Pasteurized and homogenized cows’ milk (3.5% full cream) and instant coffee powder (Nescafe, Nestlé, Milan, Italy) were purchased at a local supermarket (Reggio Emilia, Italy).

Chris Hansen’s (Denmark) commercial yogurt starter culture consisted of *Streptococcus thermophilus* and *Lactobacillus delbrueckii* ssp. *bulgaricus* in a 1:1 ratio, and this was used for the fermentation process. Chemicals and enzymes for the in vitro digestion experiments, total phenolic compounds, and antioxidant activity assays as well as phenolic standards were purchased from Sigma-Aldrich (Milan, Italy). All the solvents used in phenolic extraction and mass spectrometry analysis were purchased from BioRad (Hercules, CA, USA).

### 3.2. Preparation of Yogurts and Coffee Samples

**Figure 4:**
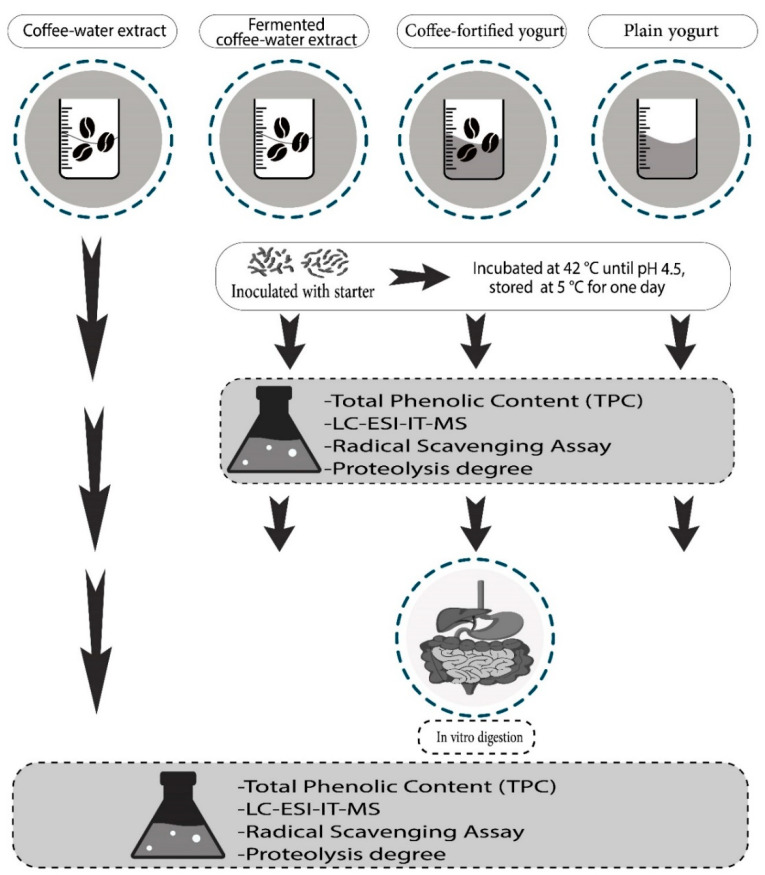
Experimental plan for the preparation and characterization of coffee samples and coffee-fortified yogurt.

The yogurt preparation and experimental design are illustrated in Figure 4. The yogurt was made according to Helal and Tagliazucchi [8] with some modifications. In each of the three replications, the pasteurized milk was mixed with 7% (*w*/*v*) sucrose and heated at 95 °C for 5 min, after which the milk was separated into two portions. One part was used for plain yogurt preparation and the other part was utilized for coffee-fortified yogurt preparation. After heating, 0.75% of instant coffee powder was added to milk for the preparation of the coffee-fortified yogurt sample. Then, both the mixtures were cooled to 45 °C and inoculated with 3% (*w*/*v*) of the yogurt starter culture YF-L811 (previously activated in pasteurized milk). Each treatment was divided into three sterile beakers and incubated at 42 °C until pH 4.5 was attained (about four hours). The yogurt samples (plain yogurt and coffee-fortified yogurt) were stored in the refrigerator at 5 °C for one day and then kept at −80 °C for the analysis.

Additionally, a coffee sample was made by substituting milk with water and treated following the same procedures reported for the coffee-fortified yogurt production (heating, inoculation, incubation, and cooling), obtaining a fermented coffee sample. Aliquots of coffee sample were withdrawn immediately after the dissolution of coffee in water (before fermentation; coffee sample) and at the end of the fermentation process (fermented coffee sample).

### 3.3. In Vitro Gastro-Intestinal Digestion

The in vitro digestion protocol INFOGEST 2.0, developed within the COST Action INFOGEST and further updated by Brodkorb et al. [72], was applied to simulate the gastro-intestinal digestion of yogurt preparations (plain yogurt and coffee-fortified yogurt), as well as of the fermented coffee sample. The simulated salivary (SSF), gastric (SGF), and intestinal (SIF) fluids were prepared following the INFOGEST 2.0 protocol. To replicate the mastication phase, homogenized samples (1 g) were well mixed with 1 mL of SSF (containing 150 U/mL of porcine α-amylase) and incubated for 2 min at 37 °C on a rotating wheel (10 rpm). To simulate the gastric phase of the digestion, 5 mL of SGF were added to the bolus. Before the addition of pepsin (2000 U/mL), the pH was corrected to 3 with HCl 6 mol/L. The gastric phase was carried out for 120 min at 37 °C on a rotating wheel (10 rpm). At the end of the incubation, 4 mL of intestinal fluid was added to the gastric digested samples, the pH was raised to 7.5 and the samples were incubated for 30 min before adding pancreatin (200 U/mL based on trypsin activity). Then, the intestinal step was carried out for an additional 120 min at 37 °C on a rotating wheel (10 rpm).

For each digestion sample, an aliquot was collected at the end of the digestion. All the digestions were performed in triplicate.

### 3.4. Samples Preparation

To remove insoluble material, samples from yogurt preparations (plain yogurt and coffee-fortified yogurt), coffee (coffee and fermented coffee) and in vitro gastro-intestinal digestions were centrifuged at 17,500× *g* for 10 min at 5 °C. After this, the clear supernatants were withdrawn and frozen at −80 °C for further analysis.

### 3.5. Determination of Total Phenolic Compounds

The concentrations of total phenolic compounds in the different samples before and after the in vitro gastro-intestinal digestion were estimated using the spectrophotometric Folin–Ciocalteu assay [73]. Gallic acid was used for the calibration curve, and the data are expressed as mg of gallic acid equivalent per 100 g of yogurt or coffee.

### 3.6. Antioxidant Activity Analysis

The antioxidant activity of samples before and after in vitro gastro-intestinal digestion was determined using three different assays.

The ABTS (2,2′-azinobis(3-ethylbenzothiazoline-6-sulphonic acid)) assay was carried out according to Re et al. [74]. Briefly, 40 μL of appropriately diluted sample were added to 1960 μL of ABTS radical cation. The mixture was then incubated at 37 °C for 10 min, and the decrease in absorbance was determined at 734 nm. The results have been expressed as mg of ascorbic acid equivalent/100 g of yogurt or coffee water extract.

The DPPH method was performed as described by Helal et al. [75]. Briefly, 200 μL of sample was added to 2 mL of a 0.1 mmol/L methanolic solution of DPPH. The absorbance was measured at 517 nm after incubation for 30 min in the dark. Ascorbic acid was used for the calibration curve and the results are expressed as mg ascorbic acid equivalent/100 g of yogurt or coffee water extract.

The ferric reducing antioxidant power (FRAP) assay was carried out according to Benzie and Strain [76]. The working FRAP reagent (WFR) was prepared by mixing acetate buffer (0.3 mol/L, pH 3.6), TPTZ solution (10 mmol/L) and FeCl_3_ solution (20 mmol/L) in a ratio of 10:1:1. Then, 100 μL of the analyzed sample was mixed with 3 mL of WFR. The absorbance at 593 nm was then recorded after 6 min of reaction. The results are expressed as mg ascorbic acid equivalent/100 g of yogurt or coffee water extract.

### 3.7. Determination of Protein Hydrolysis

The 2,4,6-trinitrobenzenesulphonic acid (TNBS) method described by Adler Nissen [77] was used for the assessment of proteolysis degree. L-leucine was used as the standard reference.

### 3.8. Identification of Individual Phenolic Compounds by Mass Spectrometry

The identification of individual phenolic compounds in coffee as well as in undigested and digested fermented coffee and coffee-fortified yogurt was carried out using a liquid chromatography electrospray ionization ion trap mass spectrometer (LC-ESI-IT-MS). The system consisted of an HPLC Agilent 1200 Series (Agilent Technologies, Santa Clara, CA, USA) coupled with an Agilent 6300 ion trap mass spectrometer. A C18 column (HxSil C18 Reversed phase, 250 × 4.6 mm, 5 μm particle size, Hamilton Company, Reno, NV, USA) was used to separate individual phenolic compounds. The chromatographic condition as well as the mass spectrometry parameters are fully described in Martini et al. [78]. The concentrations of individual phenolic compounds were determined using authentic standards.

### 3.9. Bioaccessibility Index

The bioaccessibility index (BI%) was calculated as described by Martini et al. [79] to evaluate the effect of the matrix composition on the phenolic compounds and coffee phenolic compounds released during in vitro gastro-intestinal digestion:

$$\mathrm{Bioaccessibility}\;\mathrm{Index}\;(\%)=\frac{Cd}{Cc}\times 100$$

where *Cd* is the concentration of phenolic compounds found after in vitro gastro-intestinal digestion, whereas C*c* is the concentration in the coffee sample.

### 3.10. Statistics

All data are presented as means ± SD for three replicates of each prepared sample. When multiple comparisons were made, Graph Pad prism 8.0 (GraphPad Software, San Diego, CA, USA) was used to perform a univariate analysis of variance (ANOVA) with Tukey’s post-hoc test. *p* < 0.05 was used to determine whether the differences were significant.

## 4. Conclusions

As a result of its multi-functional and health properties, yogurt is considered one of the most popularly consumed fermented dairy products. Moreover, much evidence has proven the health benefits of phenolic compounds, including coffee hydroxycinnamic acids. Therefore, phenolic compounds have been widely proposed as fortifying agents for functional food production, especially during fermented dairy products’ manufacturing. Coffee-fortified yogurt has shown a higher phenolic content (both total and individual), and therefore higher antioxidant activity, compared to plain yogurt. After in vitro digestion, the yogurt matrix had a protective effect on the phenolic compounds’ stability, with respect to the fermented coffee sample, enhancing their release in the gastro-intestinal tract. A significant increase in antioxidant activity in coffee-fortified yogurt was observed at the end of the gastro-intestinal digestion, and this was higher than in plain yogurt or fermented coffee as well. This high antioxidant activity of coffee-fortified yogurt means it can be considered a viable choice for the free radical protection of the gastro-intestinal system. In addition to the health benefits of yogurt, coffee-fortified yogurt revealed substantial coffee phenolic compound levels with high bioaccessibility and high antioxidant activity following in vitro digestion. Therefore, the manufactured coffee-fortified yogurt may combine the biological activities of the yogurt components with those of coffee phenolic compounds, and can be considered a functional food with improved health properties.

## Figures and Tables

**Table 1:** Individual phenolic compounds (µmol/100 g) identified and quantified in coffee (C), fermented coffee (FC) and coffee-fortified yogurt (CY) before and after in vitro gastro-intestinal digestion. Bioaccessibility index (BI) is the percentage ratio between the concentration in the digested sample and the concentration in the coffee water extract before digestion.

Phenolic Compounds	Before Digestion			After Digestion	
	C	FC	CY	FC	CY
*Caffeoylquinic acids*					
3-*O*-Caffeoylquinic acid	10.76 ± 0.14 ^a^	6.00 ± 0.15 ^b^	4.27 ± 0.09 ^c^	1.56 ± 0.09 ^d^	1.55 ± 0.03 ^d^
4-*O*-Caffeoylquinic acid	1.90 ± 0.02 ^b^	2.53 ± 0.35 ^a^	1.27 ± 0.01 ^c^	n.d.	1.39 ± 0.01 ^c^
5-*O*-Caffeoylquinic acid	10.32 ± 0.19 ^a^	5.15 ± 0.24 ^b^	3.50 ± 0.09 ^c^	1.77 ± 0.01 ^d^	0.51 ± 0.00 ^e^
*Total caffeoylquinic acids*	22.98 ± 0.24 ^a^	13.68 ± 0.45 ^b^	9.04 ± 0.13 ^c^	3.33 ± 0.10 ^d^	3.45 ± 0.04 ^d^
BI%				14.5	15.0
*Feruloylquinic acids*					
3-*O*-Feruloylquinic acid	4.67 ± 0.11 ^a^	2.72 ± 0.01 ^b^	1.94 ± 0.00 ^c^	1.11 ± 0.01 ^d^	4.74 ± 0.10 ^a^
4-*O*-Feruloylquinic acid	0.82 ± 0.01 ^d^	2.84 ± 0.22 ^b^	3.17 ± 0.14 ^a^	2.50 ± 0.01 ^c^	2.97 ± 0.08 ^ab^
5-*O*-Feruloylquinic acid	2.79 ± 0.09 ^a^	1.76 ± 0.18 ^c^	1.74 ± 0.05 ^c^	2.52 ± 0.01 ^b^	2.81 ± 0.10 ^a^
*Total feruloylquinic acid*	8.28 ± 0.15 ^b^	7.32 ± 0.29 ^c^	6.85 ± 0.15 ^c^	6.13 ± 0.05 ^d^	10.52 ± 0.16 ^a^
BI%				74.0	127.1
Caffeoylquinic acid-glycosides					
Caffeoylquinic acid-glycoside isomer 1	1.11 ± 0.03 ^a^	1.12 ± 0.09 ^a^	1.03 ± 0.02 ^a^	n.d.	n.d.
Caffeoylquinic acid-glycoside isomer 2	0.68 ± 0.01	n.d.	n.d.	n.d.	n.d.
Caffeoylquinic acid-glycoside isomer 3	0.80 ± 0.01	n.d.	n.d.	n.d.	n.d.
Caffeoylquinic acid-glycoside isomer 4	0.60 ± 0.02 ^c^	0.96 ± 0.03 ^a^	0.81 ± 0.01 ^b^	n.d.	n.d.
*Total caffeoylquinic acid-glycosides*	3.19 ± 0.06 ^a^	2.08 ± 0.09 ^b^	1.84 ± 0.04 ^c^	/	/
BI%				/	/
*Di-caffeoylquinic acids*					
3,4-di-*O*-caffeoylquinic acid	2.10 ± 0.02 ^a^	1.39 ± 0.00 ^b^	n.d.	n.d.	n.d.
4,5-di-*O*-caffeoylquinic acid	0.50 ± 0.00 ^b^	0.49 ± 0.00 ^b^	0.78 ± 0.02 ^a^	n.d.	n.d.
3,5-di-*O*-caffeoylquinic acid	0.45 ± 0.01	n.d.	n.d.	n.d.	n.d.
*Total dicaffeoylquinic acids*	3.05 ± 0.04 ^a^	1.88 ± 0.00 ^b^	0.78 ± 0.02 ^c^	/	/
BI%				/	/
*Others hydroxycinnamic acids*					
Caffeoylshikimic acid isomer 1	n.d.	0.12 ± 0.01	n.d.	n.d.	n.d.
Caffeoylshikimic acid isomer 2	n.d.	1.10 ± 0.02 ^a^	0.55 ± 0.01 ^b^	n.d.	n.d.
Caffeoyl hexose	n.d.	n.d.	0.59 ± 0.01	n.d.	n.d.
*Total others hydroxycinnamic acids*	/	1.22 ± 0.02 ^a^	1.14 ± 0.05 ^a^	/	/
BI%				/	/
*Total hydroxycinnamic acids*	37.50 ± 0.17 ^a^	26.18 ± 0.54 ^b^	19.65 ± 0.21 ^c^	9.46 ± 0.11 ^e^	13.97 ± 0.16 ^d^
BI%				25.2	37.3

n.d. means not detected; different superscript letters within the same row indicate that the values are significantly different (*p* < 0.05).

## Data Availability

The data presented in this study are available herein.

## Sample Availability

Samples of the compounds are available from the authors.
